# High-Polyphenol Fruit and Vegetable Consumption and Cardiovascular Disease (CVD) Risk Factors Among Adults in Jeddah, Saudi Arabia

**DOI:** 10.7759/cureus.66863

**Published:** 2024-08-14

**Authors:** Sarah N Alsharif

**Affiliations:** 1 Department of Clinical Nutrition, Faculty of Applied Medical Sciences, King Abdulaziz University, Jeddah, SAU

**Keywords:** jeddah, cvd risk, vegetables, fruits, polyphenols

## Abstract

Background: Polyphenols found in food is a potential modifiable factor in disease prevention, especially when it comes to cardiovascular diseases (CVDs). This study aimed to determine the total polyphenol intake from fruits and vegetables (FV) in patients at King of Abdulaziz University Hospital, Jeddah, Saudi Arabia, and its association with vascular risk biomarkers.

Methods: A cross-sectional study was done on 151 adult patients with at least one CVD risk factor. Data about demographics, smoking status, physical activity, height and weight, waist-to-hip ratio (WHR), waist circumference (WC), disease history, current disease (CD), and amount of polyphenol intake (mg/100 g) from rich polyphenol FV sources were collected.

Results: Of the participants, 127 (84.1%) were females, 49 (32.5%) had an age ranging from 45 to 54 years, and 110 (72.8%) were married. Of them, 54 (35.8%) had a bachelor's education, and 64 (42.4%) were employed. Moreover, 89 (59.3%) were physically inactive, 18 (11.9%) were smokers, 105 (69.5%) were obese, 116 (76.9%) had high WC, and 103 (68.2%), 109 (72.2%), and 90 (59.6%) had a family history of diabetes mellitus (DM), hypertension (HTN), and hyperlipidemia, respectively. The mean total polyphenol consumption/gm was significantly higher among older and married participants, and patients with HTN had a significantly lower mean total polyphenol consumption/gm. A significant positive correlation was found between the total polyphenol consumption/gm and participants’ age.

Conclusion: The consumption of polyphenols was associated with age, marital status, and blood pressure. Polyphenols from FV may have a preventive effect against cardiovascular illnesses. Including a range of foods high in polyphenols in a balanced diet is still a potential way to support cardiovascular health.

## Introduction

Polyphenols are frequently found in human diets. According to several studies [1,2,3], they are secondary plant metabolites that are ingested through specific plant-based diets and have potential health advantages [1]. Generally, polyphenols are organic substances made by plants, fungi, or bacteria that do not directly contribute to an organism's development or regular growth. Fruits, vegetables, grains, chocolate, tea, coffee, nuts, and herbs are sources of polyphenols [4,5].

According to Karam et al. [1], polyphenols have at least one aromatic ring structure and one or more hydroxyl groups. The types of plants have different amounts of polyphenols [6]. Polyphenols can be categorized into numerous classes based on differences in the number or placement of chemical groups within their structure [1].

Foods contain a variety of polyphenols, the most prevalent of which are phenolic acids, flavonoids, lignans, and stilbenes [5,6]. These subclasses are critical to human health, particularly in relation to cardiovascular diseases (CVDs). There are two types of phenolic acids: derivatives of cinnamic acid and derivatives of benzoic acid. With the exception of some red fruits, black radish, and onions, its edible plant content is typically relatively low [7].

The most common type of flavonoid found in food is flavonol, which is found in abundance in broccoli, leeks, onions, curly kale, and blueberries. Citrus fruits, such as grapefruit, oranges, and lemons, have high flavonoid content in their skin. Fruit (prunes and pears) contains lignans [1]. Only trace amounts of stilbenes can be detected in wine and the human diet [8].

Polyphenols have positive effects on the body's health, preventing conditions including diabetes mellitus (DM), CVD, hypertension (HTN), neurological illnesses, and some types of cancer, among others [1]. As a result, scientists are currently focusing on understanding the polyphenol's therapeutic effectiveness in the body, including its kind, average daily food consumption, and bioavailability in terms of tissue penetration, plasma concentration, mode of absorption, and routes of disposal. High intakes of polyphenols from various fruits and vegetables (FV) can reduce the chance of developing chronic illnesses [7,8,9].

Polyphenols work in the body to trap and scavenge free radicals, regulate nitric oxide, reduce leukocyte immobilization, induce apoptosis, and exhibit phytoestrogen activity, among many other beneficial effects on human health [1,9]. These protective biological properties of polyphenols have the ability to alleviate disease [1].

Consuming polyphenols has been shown to improve human health, particularly CVDs. Studies conducted in vitro and in vivo have demonstrated the effects of polyphenol-rich foods on blood pressure, vascular function, and blood lipid levels [10]. Polyphenols have the potential to lower blood pressure by enhancing endothelial-dependent vasodilation and decreasing homeostasis, which is a risk factor for CVD. Another way that polyphenols work is that they are strong antioxidants that help the body reorganize lipids, particularly in the liver [10].

Every year, 17.3 million persons with CVDs pass away worldwide. According to estimates made by the World Health Organization (WHO) in 2011, there would be 23.6 million of these people by 2030 [11]. According to Ahmed et al. [12] and Alshaikh et al. [11], there is a significant prevalence of CVD risk factors in Saudi Arabia, such as smoking, obesity, dyslipidemia, DM, HTN, and physical inactivity. Saudi women smoke at a rate of 2.5-9.1%, whereas males smoke at a relatively high rate of 11.6-52.3% [12]. A high percentage (47.5%) of people have HTN as of late [12]. According to Alzahrani et al. [13], 23.9% of people have diabetes, 35.1% of men and 30.1% of women are overweight, 34.8% of men and 35.6% of women are obese, and 60.1% of men and 72.9% of women are physically inactive. Therefore, following a healthy diet such as the Mediterranean diet and the Dietary Approaches to Stop Hypertension (DASH) are the first steps to managing the CVD risk factors. Both diets are recommended to consume five servings per day of FV. Thus, a polyphenol-rich diet may help to reduce CVD risk [14]. Saudi's latest National Health Survey data showed that 97.2% of adults consume fewer than five portions/day of FV [15]. 

Most of the previous studies assessed FV consumption per day among females or estimated one specific type of polyphenols in one type of fruit as dates [11,15]. One of the important studies was a case-control study done in 2007 to assess the effects of dietary flavonoid intake in Saudi patients with coronary heart disease (CHD) [16]. According to the study, CHD patients consumed substantially fewer FV than the control group. Patients with congestive heart failure had considerably greater serum lipid levels than the control group. In a normal Saudi diet, tea, fruits (such as apples), vegetables (like onions), and chocolate were the main sources of flavonoids. When CHD patients were compared to the controls, their consumption of flavonoids, β-carotene, and vitamin C was significantly lower [16].

There is limited data about total polyphenol intake from FV among adults in Saudi Arabia. To the best of our knowledge, this is the first study to determine the consumption of total polyphenol intake from FV in male and female patients from different clinics at King of Abdulaziz University Hospital, Jeddah, Saudi Arabia, and to assess the association between total polyphenols from FV and cardiovascular risk biomarkers in adults.

## Materials and methods

Study design, setting, and time

A cross-sectional study was done at King Abdulaziz University Hospital, Jeddah, Saudi Arabia, from January to March 2020.

Study participants

The inclusion criteria were adult patients with at least one risk factor of CVD, ≥18 years, and Saudi nationals. Patients with known CVD, i.e., ischemic heart disease, stroke history, and other serious diseases, were excluded from the study.

Sample size

The total sample size was 151 participants, which was calculated using the G*Power software (Heinrich-Heine-Universität Düsseldorf, Germany), with 95% power, 5% significance, and 2% effect size.

Data collection

Patients were recruited to complete the structured questionnaire, and a brief description of the study was explained to them. The burden time was estimated to take about four minutes, and questions were answered verbally by the interviewees. At the beginning of the interview, the interviewer introduced herself to the participants, and she informed the patients about the expected duration of the interview. Afterward, 16 close-ended questions were administered, and at the end of the interview, a series of questions assessed sociodemographic characteristics. 

The initial questionnaire was designed in English and translated to Arabic by two experts and then back-translated into English by another two independent experts to ensure the validity of the constructs to be assessed. The final questionnaire was divided into three sections. The first section inquired about demographic data (e.g., gender, age, marital status, educational levels, employment condition, smoking status, physical activity, height, and weight). The second section enquired about polyphenols from FV intake. For an accurate report of FV intake, pictures of FV were used. The last section inquired about disease history and current diseases (e.g., family history of DM, HTN, and hyperlipidemia).

The weight and height were taken from the patient's recall and from filling up the questionnaire, the body mass index (BMI) was calculated as weight (kg) divided by height (m) squared (kg/m^2^). According to the anthropometric reference parameters for adults, the prevalence of underweight, normal weight, overweight, and obesity are BMI < 18 kg/m^2^, 18-24.9 kg/m^2^, 25-29.9 kg/m^2^, and >=30 kg/m^2^. In addition, the WHR and WC were measured during the interview by the investigator using a meter.

The amount of polyphenols intake (mg/100 g) from rich-polyphenol FV sources included in the study questionnaire and biomarkers of CVD risk factors were taken from patients' recall and form filling the questionnaire. The daily servings of a specific fruit/vegetable (containing polyphenols) were self-reported by the participant in the questionnaire, and the options were zero and six servings. The total servings of all fruits/vegetables (containing polyphenols) per day was calculated. The total polyphenols in (mg) consumed per day was calculated by the conversion of servings into weight (grams) and then applying the concentration of polyphenols per 100 grams of the fruit/vegetable. The conversion of servings into weight relied on the Nutrition Value website (https://www.nutritionvalue.org/) [17].

The definition of servings was taken from the National Health Service (NHS) website, and the polyphenol concentration was taken from the study of Pierre Brat et al. (2007). Table 1 summarizes all of the information that we relied on from these sources.

**Table 1:** Serving definition, serving weight, and polyphenol concentration of fruits/vegetables N.B.: We considered that one stalk contains eight to nine sticks (this was specifically taken from https://www.howmuchisin.com/produce_converters/celery)[18].

Fruit / vegetable	Serving definition	Serving weight (mg)	Polyphenol (mg) per 100 g of the fruit / vegetable
Strawberry	7 strawberries	84	263.8
Artichoke heart	½ to 1 whole artichoke	96	321.3
Lychee	6 lychees	60	222.3
Parsley	10 sprigs	10	280.2
Grape	(16 grapes, 1/2 cup)	78.4	195.5
Brussels sprouts	8 sprouts	152	257.1
Apricot	(1 fresh, 1/2 cup canned. or 5 dried)	35	179.8
Shallot	(1 large shallot, 1/2 cup minced or sliced)	80	104.1
Apple	1 fruit	182	179.1
Broccoli	1/2 cup	45.5	98.9
Dates	5 dates	40	99.3
Celery	2 to3 sticks*	11.8^1^	84.7
Cherry	14 cherries	114.8	94.3
Onion	1 slice	14	76.1
Pear	1 fruit	178	69.2
Asparagus	5 asparagus spears	80	14.5
White nectarine	1 medium	142	72.7
Eggplants	One-third a piece	182.7	65.6
Passion fruit	5 to 6 passion fruit	99	71.8
Garlic	1 to 2 cloves	4.5	59.4

Ethical considerations

The ethical approval to conduct this study was obtained from the Unit of Biomedical Ethics Research Committee at King Abdulaziz University (Reference No. 85-20). The patients signed a consent form to participate in this study.

Data analysis

Data were statistically analyzed using the IBM SPSS Statistics for Windows, version 26.0 (released 2019, IBM Corp., Armonk, NY). To investigate the association between the variables, the chi-squared test (χ2) was applied to qualitative data that were expressed as numbers and percentages. Quantitative data were expressed as mean and standard deviation (mean ± SD), where Mann-Whitney and Kruskal-Wallis tests were applied for non-parametric variables. Correlation analysis using the Spearman’s test was done, and a p-value of <0.05 was considered statistically significant.

## Results

Participant demographics

Of the studied 151 participants, 127 (84.1%) were females, 49 (32.5%) had an age ranging from 45 to 54 years, and 110 (72.8%) were married. Of them, 54 (35.8%) had a college degree (bachelor's) in education, 68 (45%) were housewives, and 64 (42.4%) were employed. Almost one-third of the participants (43, 28.5%) had a monthly income of more than 8000 SR (Table 2).

**Table 2:** Distribution of the studied participants according to their demographic characteristics (n = 151) N.B.: 1 = Sum of frequencies is not exactly 151 (just slightly less, by one or two participants) due to missing values/information (i.e., information not provided by the participant).

Variable	No. (%)
Gender	
Male	24 (15.9)
Female	127 (84.1)
Age	
18-24	6 (4)
25-34	22 (14.6)
35-44	47 (31.1)
45-54	49 (32.5)
55-64	22 (14.6)
65+	5 (3.3)
Marital status^1^	
Single	41 (27.2)
Married	110 (72.8)
Education	
Illiterate	11 (7.3)
Elementary school	18 (11.9)
Intermediate school	11 (7.3)
High school	52 (34.4)
Diploma	3 (2)
College degree (bachelor)	54 (35.8)
Doctor of Philosophy (PhD)	2 (1.3)
Employment status	
Employed (government)	46 (30.5)
Employed (private sector)	18 (11.9)
Student	3 (2)
Unemployed	10 (6.6)
Housewife	68 (45)
Retired	6 (4)
Monthly income (SAR)	
<2000	32 (21.2)
2000-3000	39 (25.8)
4000-5000 (median)	14 (9.3)
6000-7000	23 (15.2)
>8000	43 (28.5)

Most of the participants were physically inactive (89, 59.3%), and 18 (11.9%) were smokers. The mean BMI was 33.35 ± 6.44 kg/m^2^, and the majority (105, 69.5%) were obese. The mean WC for males and females was 104.04 ± 16.29 cm and 100.26 ± 13.93 cm respectively, with 83 (55%) having a high WC and 33 (21.9%) having a very high one. The mean hip circumference was 114.18 ± 16.52 cm, with 54 (35.7%) of the participants having a hip circumference > 120 cm. The mean waist-hip ratio was 0.89 ± 0.12, with 127 (84.1%) of the participants having a waist-hip ratio >0.8. Most of the participants (103, 68.2%) had a family history of DM, 109 (72.2%) had a family history of HTN, and 90 (59.6%) had a family history of hyperlipidemia. The most common CD was HTN (95, 63.8%), followed by DM (40, 26.8%) (Table 3).

**Table 3:** Distribution of the participants according to the cardiovascular risk biomarkers (n = 151) N.B.: 1 = sum of frequencies is not exactly 151 (just slightly less, by one or two participants) due to missing values/information (i.e., information not provided by the participant). BMI = body mass index, SD = standard deviation, WC = waist circumference, cm = centimeter, DM = diabetes mellitus, HTN = hypertension, CD = current disease.

Variable	(No.) (%)
Physical activity^1^	
Yes	61 (40.7)
No	89 (59.3)
Smoking	
Never smoked	133 (88.1)
Smoker	18 (11.9)
BMI	
Normal	8 (5.3)
Overweight	38 (25.2)
Obese	105 (69.5)
BMI (Mean ± SD)	33.35 ± 6.44
WC (cm)	
Very low	10 (6.6)
Low	25 (16.6)
High	83 (55)
Very high	33 (21.9)
WC (cm) (Mean ± SD) for males	104.04 ± 16.29
WC (cm) (Mean ± SD) for females	100.26 ± 13.93
Hip (cm)	
< 100	22 (14.6)
100-109.9	29 (19.2)
110-119.9	46 (30.5)
120-129.9	31 (20.5)
130-139.9	18 (11.9)
140+	5 (3.3)
Hip (cm) (Mean ± SD)	114.18 ± 16.52
Waist-hip ratio	
>0.8	127 (84.1)
<0.8	24 (15.9)
Waist-hip ratio (Mean ± SD)	0.89 ± 0.12
Family history of DM	
Yes	103 (68.2)
No	48 (31.8)
Family history of HTN	
Yes	109 (72.2)
No	42 (27.8)
Family history of hyperlipidemia	
Yes	(90) (59.6)
No	(61) (40.4)
Most prominent CD^1^	
Hypertension	95 (63.8)
Diabetes	40 (26.8)
Obese	13 (8.7)
Hyperlipidemia	1 (0.7)

Table 4 shows that three fruits/vegetables have high daily consumption (median daily portions = 5), namely, garlic, onion, and dates. A medium consumption of two to three portions was seen in apples, brussels sprouts, grapes, and parsley. Low consumption of one portion was seen in cherry, pear, eggplants, passion fruit, apricot, and white nectarine. Almost no consumption (median = 0) was seen in shallot, strawberry, broccoli, celery, artichoke heart, lychee, and asparagus.

**Table 4:** Median (and IQR) for the daily consumption fruits/vegetables portions N.B.: 1 = Median participant number of consumed servings for each fruit/vegetable not normally distributed.

Fruit / vegetable	Median^1^ (IQR) of portions/day
Garlic	5 (4-5)
Onion	5 (3-5)
Dates	5 (2-5)
Apple	3 (1-5)
Brussels sprout	3 (0-5)
Grape	2 (1-4)
Parsley	2 (0-5)
Cherry	1 (0-3)
Pear	1 (0-3)
Eggplants	1 (0-3)
Passion fruit	1 (0-3)
Apricot	1 (0-2)
White nectarine	1 (0-2)
Shallot	0 (0-2)
Strawberry	0 (0-1)
Broccoli	0 (0-1)
Celery	0 (0-1)
Artichoke heart	0 (0-0)
Lychee	0 (0-0)
Asparagus	0 (0-0)

Table 5 shows that the mean total polyphenols consumption/gm was significantly higher among participants with older age (65+ years) and among married participants (p ≤ 0.05). On the other hand, a non-significant relationship was found between the mean polyphenol consumption/gm and participants' gender, educational level, employment status, or monthly income (p ≥ 0.05).

**Table 5:** Relationship between mean polyphenol consumption/gm and participants demographics (n = 151) N.B.: 1 = Sum of frequencies is not exactly 151 (just slightly less, by one or two participants) due to missing values/information (i.e., information not provided by the participant).

Variable	Total polyphenol consumption / gm	Test	p-value
	Mean ± SD		
Gender			
Male	3.59 ± 1.88	1.05	0.295
Female	4 ± 1.72		
Age			
18-24	2.48 ± 1.66	3.01	0.013
25-34	3.12 ± 1.52		
35-44	3.82 ± 1.95		
45-54	4.4 ± 1.46		
55-64	4.11 ± 1.76		
65+	4.82 ± 1.63		
Marital status^1^			
Single	3.19 ± 1.97	3.27	0.001
Married	4.21 ± 1.58		
Education			
Illiterate	3.97 ± 1.96		
Elementary school	4.29 ± 1.3	1.3	0.258
Intermediate school	3.89 ± 1.95		
High school	4.34 ± 1.76		
Diploma	3.72 ± 3.01		
College degree (bachelor's)	3.45 ± 1.67		
Doctor of Philosophy (PhD)	3.45 ± 2.41		
Employment status			
Employed (government)	3.96 ± 1.76	0.98	0.429
Employed (private sector)	3.65 ± 1.74		
Student	2.46 ± 1.83		
Unemployed	3.28 ± 1.58		
Housewife	4.13 ± 1.77		
Retired	4.05 ± 1.51		
Monthly income (SAR)			
<2000	4.25 ± 1.87	0.53	0.709
2000-3000	4 ± 1.65		
4000-5000 (median)	3.52 ± 1.84		
6000-7000	3.79 ± 1.94		
>8000	3.83 ± 1.64		

**Figure 1:**
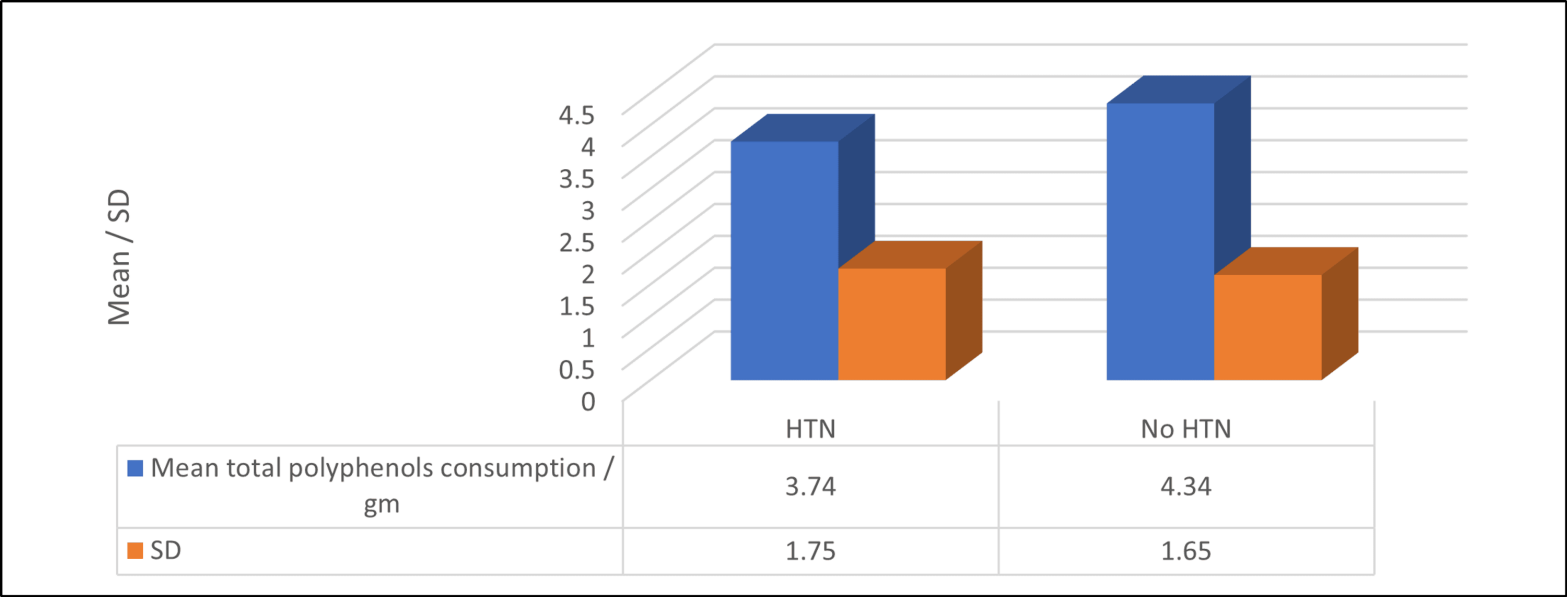
Relationship between mean polyphenol consumption/gm and HTN prevalence (n = 151) N.B.: SD = standard deviation, HTN = hypertension

As for cardiovascular risk biomarkers, Table 6 and Figure 1 demonstrate that patients with HTN had a significantly lower mean total polyphenol consumption/gm compared to non-HTN participants (3.74 ± 1.75 vs. 4.34 ± 1.65) (p ≤ 0.05). On the other hand, a non-significant relationship was found between the mean polyphenol consumption/gm and all other cardiovascular risk biomarkers or anthropometric measurements (p ≥ 0.05).

**Table 6:** Relationship between mean polyphenol consumption/gm and cardiovascular risk biomarkers (n = 151) N.B.: 1 = Sum of frequencies is not exactly 151 (just slightly less, by one or two participants) due to missing values/information (i.e., information not provided by the participant). BMI = body mass index, SD = standard deviation, WC = waist circumference, cm = centimeter, DM = diabetes mellitus, HTN = hypertension, CD = current disease.

Variable	Total polyphenol consumption/gm	Test	p-value
	Mean ± SD		
Physical activity^1^			
Yes	4.09 ± 1.77	0.32	0.99
No	3.8 ± 1.75		
Smoking			
Never smoked	3.98 ± 1.71	0.89	0.37
Smoker	3.58 ± 2.05		
BMI Mean = 33.35, SD = 6.44			
Normal	4.11 ± 1.63	0.04	0.957
Overweight	3.92 ± 1.97		
Obese	3.92 ± 1.69		
WC (cm) Males: Mean = 104.04, SD = 16.29. Females: Mean = 100.26, SD = 13.93			
Very low	4.16 ± 1.03	0.45	0.713
Low	3.66 ± 1.69		
High	3.89 ± 1.81		
Very high	4.16 ± 1.83		
Hip (cm) Mean = 114.18, SD = 16.52			
<100	4.22 ± 1.34	0.4	0.845
100-109.9	3.82 ± 1.62		
110-119.9	3.71 ± 1.91		
120-129.9	4.06 ± 1.83		
130-139.9	4.17 1±.91		
140+	3.68 ± 1.79		
Waist-hip ratio Mean = 0.89, SD = 0.12			
>0.8	3.82 ± 1.75	1.8	0.073
<0.8	4.52 ± 1.67		
Family history of DM			
Yes	4.05 ± 1.68	1.18	0.236
No	3.68 ± 1.87		
Family history of HTN			
Yes	4.01 ± 1.75	0.89	0.371
No	3.72 ± 1.74		
Family history of hyperlipidemia			
Yes	3.96 ± 1.64	0.28	0.778
No	3.88 ± 1.91		
Most prominent CD^1^			
Hypertension			
No	4.34 ± 1.65	2.03	0.044
Yes	3.74 ± 1.75		
Diabetes			
No	3.81 ± 1.74	1.76	0.079
Yes	4.37 ± 1.67		
Obese			
No	3.92 ± 1.74	0.83	0.406
Yes	4.34 ± 1.66		

Table 7 shows that a significant positive correlation was found between the total polyphenol consumption/gm and participants’ age (r = 0.26, p-value = 0.001). On the other hand, a non-significant positive correlation was found between the total polyphenol consumption/gm and BMI and hip circumference (p ≥ 0.05). A non-significant negative correlation was found between the total polyphenols consumption/gm and WC (p ≥ 0.05).

**Table 7:** Pearson’s correlation analysis between the total polyphenol consumption/gm and participants’ age, body mass index, and anthropometric measurements N.B.: BMI = body mass index, WC = waist circumference

Variable	Total polyphenol consumption/gm	
	r	p-value
Age	0.26	0.001
BMI	0.05	0.527
WC	-0.01	0.892
Hip	0.04	0.619
Waist-hip ratio	-0.07	0.38

## Discussion

The current cross-sectional study conducted in Saudi Arabia investigated the consumption of total polyphenol intake from the 20 most consumed individual polyphenol FV in patients with at least one risk factor of CVD. Moreover, the associations between FV polyphenol intake and sociodemographic and lifestyle characteristics were examined.

The present study observed that three fruits/vegetables have high daily consumption (median daily portions = 5), namely, garlic, onion, and dates. However, the participants predominantly consumed a medium consumption (median portions is two to three), which may be seen in apples, brussels sprouts, grapes, and parsley. A low consumption (median portions = 1) may be seen in cherry, pear, eggplants, passion fruit, apricot, and white nectarine, while almost no consumption (median = 0) may be seen in shallot, strawberry, broccoli, celery, artichoke heart, lychee, and asparagus.

This result is consistent with other studies that showed Saudi Arabians consume relatively little FV. The majority of women (97%) in research evaluating FV consumption among Saudi women reported consuming one to three servings of FV daily [15]. Moreover, two other studies revealed that Saudis did not follow the WHO's advice of consuming FV. According to the initial survey, 87.47% of participants did not meet their standards [19].

Data from 10,735 participants in another study revealed that they consumed fewer than two servings of FV daily [20]. Furthermore, the research revealed that a mere 22% of the subjects had ≥5 servings daily [21]. On the other hand, FV consumption was reported to be larger than or equivalent to five servings in another study that evaluated FV consumption among Saudi students [22].

Our findings showed a strong positive correlation between consuming FV high in polyphenols and growing older. This is in line with other research that examined FV intake patterns and discovered that while FV consumption increases with age across all population groups, it is highest among the retired [23]. In addition, a Malaysian study found that fruit consumption increased with the age of the home head [24].

Studies carried out in Turkey revealed a similar association between an individual's age and the likelihood that they will consume FV [23]. On the other hand, a different study discovered no appreciable variations in the consumption of FV among various age groups [24]. In Malaysia, adults' fruit intake habits were significantly influenced by their age [25]. Our study's results were consistent with those of earlier studies that revealed that consumption of flavonoids, stilbenes, lignans, and other polyphenols rose with age. By contrast, the consumption of total polyphenols and phenolic acids was shown to peak between the ages of 45 and 54 [26]. However, compared to the young and middle-aged groups, the elderly, especially the elderly women, had considerably lower flavonoid intakes in another cross-sectional investigation [27]. Furthermore, it is typical for elderly individuals to consume fewer polyphenols than younger individuals [27,28].

According to our research, married persons consume more FV polyphenols than single adults. This finding is in accordance with a longitudinal study that included 11,577 adult UK participants and revealed that men who were single, separated, or divorced had significantly lower intakes of FV. In addition, women who remained unmarried or got separated or divorced had decreases in their intake of several vegetables [29]. A different study conducted in Malaysia also discovered that people's eating patterns of FV were significantly influenced by their marital status [25].

In addition, the frequency of FV intake is comparatively lower among singles and households without children, according to data from the Canadian Community Health Survey, which includes 93,719 persons [30]. Nonetheless, a cross-sectional investigation including 894 Moroccan women discovered that the consumption of FV was unrelated to marital status [31], while a different study discovered no discernible variation in FV consumption based on marital status [24].

Compared to people with different marital circumstances, married people are more likely to eat FV, and they also tend to eat more vegetables overall [23]. Married participants were more likely to eat five or more servings of fruit and/or vegetables in a typical week, according to a Ugandan study [32]. Additional research has confirmed this degree of correlation [29]. Shared meal preparation, support from spouses, and social norms that encourage healthier eating can all be advantageous to married people. Moreover, research indicates that social support in married relationships may have a good effect on dietary decisions, such as increased intake of FV [33,34].

According to our research, BP and the consumption of FV polyphenols are significantly correlated. This outcome is in line with recent research that found that in a high-risk group for CVDs, a larger dietary intake of polyphenols reduced both systolic and diastolic blood pressure [35]. Compared to the control group, patients with CHD consumed less flavonoids [16]. Research has also shown that increasing the number of FV consumed can lower BP and improve microvascular function, both of which are associated with a lower risk of CVDs [36].

Thirty-five colorectal cancer patients participated in the trial, which took place five to 35 days prior to surgery. ↓LBP levels following daily ingestion of pomegranate extract rich in punicalagin (poly) phenol [37]. Healthy individuals participated in a second double-blind RCT (n = 20 in each arm). Acute (two hours) and chronic (one month) in length acute (two hours) and daily studies revealed that a one-month supply of wild blueberries (11 grams per day, comprising 150 mg of anthocyanins) significantly reduced 24-hour ambulatory blood pressure [38].

Certain fruits, like pomegranates, purple grapes, and berries, have been shown to have high quantities of flavonols, anthocyanins, and procyanidins, which may help lower CVD risk factors, especially blood pressure [10]. Research involving humans suggests that eating foods high in polyphenols can lower BP [28].

Numerous research, including one done at Saudi Aramco Hospital in the Kingdom of Saudi Arabia, have discovered a strong correlation between polyphenols and the risk of CVD. Patients with CHD were chosen for this study from both inpatient and outpatient clinics. Blood samples were taken, and use case and control studies were conducted to describe portion sizes and FV quantities as volumes. Ultimately, the results indicated that increasing the amount of flavonoids consumed may help lower blood lipid levels because of its biomarker for antioxidants [16]. Another study discovered that the polyphenols in pomegranate juice, which are abundant in Saudi and Egyptian diets, greatly reduce oxidative stress and prevent LDL oxidation. This research acquired ripe Saudi pomegranate fruits from several markets in Saudi Arabia and was used in a six-month investigation on experimental animals, during which blood samples were taken [39].

The consumption of polyphenols from FV and the WHR exhibited a trend in the current study, which is consistent with previous research showing a substantial link between flavonol intake and WHR [16]. A different study found that while there were no significant changes in hip circumference, there was a substantial reduction in waist circumference at days 60 and 90 after polyphenol intakes [40].

Increased consumption of flavonoids and total polyphenols was inversely correlated with BMI. Other MetS components, such as WHR, did not consistently show any relationships [35]. After consuming 30 g of mixed nuts daily for 12 weeks, there was an inverse correlation found between basal abdominal adiposity (WC, WHR) and Uro-A glucuronide, which is derived from complex polyphenols that are plentiful in diet [41].

Our research helped to establish a unique standard for the concentration of polyphenols in FV. The findings may serve as the main proof linking FV rich in polyphenols and CVD risk factors. This study may help with clinical studies, raising public awareness of polyphenol's benefits, and promoting polyphenol's reputation as a preventive agent against CVD risk factors. This research's face-to-face interviews with participants to lessen bias and queries about misinterpreted or missing data are among the strengths. The use of visual aids to depict FV quantities was another noteworthy strength that helped to avoid misconceptions over serving sizes. Furthermore, it was deployed in multiple clinics inside a single Jeddah hospital, selecting adult males and females aged 18 years and above who had at least one CVD risk factor. The FV that made up the study (date, apple, passion fruit, onion, garlic, and parsley) were selected due to their high intakes of the most often consumed individual polyphenols (FV). In addition, fruits accounted for 45% of the food sources of flavonoids in MED nations [26]. Fruits like apples and vegetables like onions are the main sources of flavonoids in a normal Saudi diet [16]. Furthermore, because they are not seasonal FV, they were also simple to locate and eat.

Limitations

The primary limitation of the study is the potential recall bias, particularly in assessing behaviors, such as FV consumption. In addition, the study was based on a questionnaire instead of a more accurate design like a case-control study that could have a recall bias. Other limitations include short duration and small sample size.

## Conclusions

This study reports positive significant associations between polyphenol consumption from FV and increased age, marital status, and BP. There is a positive trend between polyphenol consumption from FV and WHR in adults. These results suggest that polyphenols from FV may play a protective role against CVD through their antioxidant, anti-inflammatory, and other bioactive properties. However, while the evidence is promising, further research, including well-designed randomized controlled trials and mechanistic studies, is needed to confirm these findings and elucidate the optimal sources and amounts of polyphenols for BP management. Incorporating a variety of polyphenol-rich foods into a balanced diet remains a promising strategy for promoting cardiovascular health, including hypertension prevention and management.

## References

[1] Karam J, Bibiloni MD, Tur JA (2018). Polyphenol estimated intake and dietary sources among older adults from Mallorca Island. PLoS One.

[2] Pandey KB, Rizvi SI (2009). Plant polyphenols as dietary antioxidants in human health and disease. Oxid Med Cell Longev.

[3] Wang Y, Liu J, Chen F, Zhao G (2013). Effects of molecular structure of polyphenols on their noncovalent interactions with oat β-glucan. J Agric Food Chem.

[4] Manach C, Mazur A, Scalbert A (2005). Polyphenols and prevention of cardiovascular diseases. Curr Opin Lipidol.

[5] Tangney CC, Rasmussen HE (2013). Polyphenols, inflammation, and cardiovascular disease. Curr Atheroscler Rep.

[6] Mendonça RD, Carvalho NC, Martin-Moreno JM (2019). Total polyphenol intake, polyphenol subtypes and incidence of cardiovascular disease: The SUN cohort study. Nutr Metab Cardiovasc Dis.

[7] Manach C, Scalbert A, Morand C, Rémésy C, Jiménez L (2004). Polyphenols: food sources and bioavailability. Am J Clin Nutr.

[8] Sun B, Ribes AM, Leandro MC (2006). Stilbenes: Quantitative extraction from grape skins, contribution of grape solids to wine and variation during wine maturation. Analytica Chimica Acta.

[9] Actis-Goretta L, Ottaviani JI, Fraga CG (2006). Inhibition of angiotensin converting enzyme activity by flavanol-rich foods. J Agric Food Chem.

[10] Chong MF, Macdonald R, Lovegrove JA (2010). Fruit polyphenols and CVD risk: a review of human intervention studies. Br J Nutr.

[11] Alshaikh MK, Filippidis FT, Baldove JP, Majeed A, Rawaf S (2016). Women in Saudi Arabia and the prevalence of cardiovascular risk factors: a systematic review. J Environ Public Health.

[12] Ahmed AM, Hersi A, Mashhoud W, Arafah MR, Abreu PC, Al Rowaily MA, Al-Mallah MH (2017). Cardiovascular risk factors burden in Saudi Arabia: the Africa Middle East Cardiovascular Epidemiological (ACE) study. J Saudi Heart Assoc.

[13] Alzahrani AM, Albakri SB, Alqutub TT, Alghamdi AA, Rio AA (2019). Physical activity level and its barriers among patients with type 2 diabetes mellitus attending primary healthcare centers in Saudi Arabia. J Family Med Prim Care.

[14] Medina-Remón A, Casas R, Tressserra-Rimbau A (2017). Polyphenol intake from a Mediterranean diet decreases inflammatory biomarkers related to atherosclerosis: a substudy of the PREDIMED trial. Br J Clin Pharmacol.

[15] Alshaikh MK, Rawaf S, Quezada-Yamamoto H (2018). Cardiovascular risk and fruit and vegetable consumption among women in KSA; a cross-sectional study. J Taibah Univ Med Sci.

[16] Alsaif MA, Khan LA, Alhamdan AA, Alorf S, Al-Othman AM, Alawami S (2007). Effects of dietary flavonoids intake in Saudi patients with coronary heart disease. J Family Community Med.

[17] (2024). Nutrition value. Find nutritional value of a product. https://www.nutritionvalue.org/.

[18] (2024). Produce converter. https://www.howmuchisin.com/produce_converters/celery.

[19] Alsunni AA, Badarc A (2015). Fruit and vegetable consumption and its determinants among Saudi university students. J Taibah Univ Med Sci.

[20] Mokdad A, El Bcheraoui C, Basulaiman M (2013). Fruit and vegetable consumption among adults in Saudi Arabia. Dove Med Press.

[21] Al-Otaibi HH (2013). The pattern of fruit and vegetable consumption among Saudi university students. Glob J Health Sci.

[22] Elsoadaa S, Abdelhafez A, Rabeh N (2013). Consumption of fruits and vegetables among Umm Al- Qura University students in Makkah, Saudi Arabia: A cross -section study. Life Sci J.

[23] Küçük N, Urak F, Bilgic A, Florkowski WJ, Kiani AK, Özdemir FN (2023). Fruit and vegetable consumption across population segments: evidence from a national household survey. J Health Popul Nutr.

[24] Asli K (2020). Sociodemographic factors as determinants of fruit and vegetable consumption in Malaysia. Jurnal Sains Kesihatan Malaysia.

[25] Othman KI, Ab Karim MS, Karim R (2012). Factors influencing fruits and vegetables consumption behaviour among adults in Malaysia. J Agribus Mark.

[26] Zamora-Ros R, Knaze V, Rothwell JA (2016). Dietary polyphenol intake in Europe: the European Prospective Investigation into Cancer and Nutrition (EPIC) study. Eur J Nutr.

[27] Zujko ME, Witkowska AM, Waśkiewicz A, Mirończuk-Chodakowska I (2015). Dietary antioxidant and flavonoid intakes are reduced in the elderly. Oxid Med Cell Longev.

[28] Grosso G, Godos J, Currenti W (2022). The effect of dietary polyphenols on vascular health and hypertension: current evidence and mechanisms of action. Nutrients.

[29] Vinther JL, Conklin AI, Wareham NJ, Monsivais P (2016). Marital transitions and associated changes in fruit and vegetable intake: findings from the population-based prospective EPIC-Norfolk cohort, UK. Soc Sci Med.

[30] Azagba S, Sharaf MF (2011). Disparities in the frequency of fruit and vegetable consumption by socio-demographic and lifestyle characteristics in Canada. Nutr J.

[31] Landais E, Bour A, Gartner A, McCullough F, Delpeuch F, Holdsworth M (2015). Socio-economic and behavioural determinants of fruit and vegetable intake in Moroccan women. Public Health Nutr.

[32] Kabwama SN, Bahendeka SK, Wesonga R, Mutungi G, Guwatudde D (2019). Low consumption of fruits and vegetables among adults in Uganda: findings from a countrywide cross-sectional survey. Arch Public Health.

[33] Conklin AI, Forouhi NG, Surtees P, Khaw KT, Wareham NJ, Monsivais P (2014). Social relationships and healthful dietary behaviour: evidence from over-50s in the EPIC cohort, UK. Soc Sci Med.

[34] Carbonneau E, Lamarche B, Robitaille J (2019). Social support, but not perceived food environment, is associated with diet quality in French-speaking Canadians from the PREDISE study. Nutrients.

[35] Wisnuwardani RW, De Henauw S, Forsner M (2020). Polyphenol intake and metabolic syndrome risk in European adolescents: the HELENA study. Eur J Nutr.

[36] Woodside JV, Young IS, McKinley MC (2013). Fruit and vegetable intake and risk of cardiovascular disease. Proc Nutr Soc.

[37] González-Sarrías A, Núñez-Sánchez MA, Ávila-Gálvez MA (2018). Consumption of pomegranate decreases plasma lipopolysaccharide-binding protein levels, a marker of metabolic endotoxemia, in patients with newly diagnosed colorectal cancer: a randomized controlled clinical trial. Food Funct.

[38] Rodriguez-Mateos A, Istas G, Boschek L (2019). Circulating anthocyanin metabolites mediate vascular benefits of blueberries: insights from randomized controlled trials, metabolomics, and nutrigenomics. J Gerontol A Biol Sci Med Sci.

[39] BinMowyna M, Binobead M, Badr N (2019). Effect of Saudi and Egyptian pomegranate polyphenols in regulating the activity of PON1, PON2 and lipid profile for preventing coronary heart disease. J Regen Bio Med.

[40] Martínez-Rodríguez A, Martínez-Olcina M, Vicente-Martínez M (2024). Effectiveness of a polyphenol-enriched blend on weight management and metabolic syndrome-related parameters in healthy overweight adults. App Sci.

[41] Mora-Cubillos X, Tulipani S, Garcia-Aloy M, Bulló M, Tinahones FJ, Andres-Lacueva C (2015). Plasma metabolomic biomarkers of mixed nuts exposure inversely correlate with severity of metabolic syndrome. Mol Nutr Food Res.

